# Reflections on the challenges of conducting an international multicentre randomized controlled trial of balance training in addition to pulmonary rehabilitation and its impact on fall incidence in people with COPD

**DOI:** 10.1186/s13063-024-08251-1

**Published:** 2024-07-17

**Authors:** Anastasia N. L. Newman, Marla K. Beauchamp, Cindy Ellerton, Roger Goldstein, Jennifer A. Alison, Gail Dechman, Kimberley J. Haines, Samantha L. Harrison, Anne E. Holland, Annemarie L. Lee, Alda Marques, Lissa Spencer, Michael K. Stickland, Elizabeth H. Skinner, Pat G. Camp, Michelle E. Kho, Dina Brooks

**Affiliations:** 1 School of Rehabilitation Science, Faculty of Health Science, McMaster University, Hamilton, ON Canada; 2 Department of Respiratory Medicine, West Park Healthcare Centre, Toronto, ON Canada; 3 Department of Physical Therapy, Faculty of Medicine, University of Toronto, Toronto, ON Canada; 4 Rehabilitation Sciences Institute, School of Graduate Studies, University of Toronto, Toronto, ON Canada; 5 Department of Medicine, Faculty of Medicine, University of Toronto, Toronto, ON Canada; 6 School of Health Sciences, Faculty of Medicine and Health, University of Sydney, Sydney, NSW Australia; 7 School of Physiotherapy, Faculty of Health, Dalhousie University, Halifax, NS Canada; 8 Physiotherapy Department, Western Health, Department of Critical Care, The University of Melbourne, Melbourne, VIC Australia; 9 School of Health and Life Sciences, Teesside University, Middlesbrough, UK; 10 Department of Physiotherapy, Alfred Health, Melbourne, VIC Australia; 11 Respiratory Research, Monash University, Melbourne, VIC Australia; 12 Institute for Breathing and Sleep, Melbourne, VIC Australia; 13 Department of Physiotherapy, School of Primary and Allied Health Care, Monash University, Melbourne, VIC Australia; 14 Lab3R-Respiratory Research and Rehabilitation Laboratory, School of Health Sciences (ESSUA) and Institute of Biomedicine (iBiMED), University of Aveiro, Aveiro, Portugal; 15 Department of Physiotherapy, Royal Prince Alfred Hospital, Camperdown, NSW Australia; 16 Division of Pulmonary Medicine, Faculty of Medicine and Dentistry, University of Alberta, Edmonton, AB Canada; 17 G.F. MacDonald Centre for Lung Health, Covenant Health, Edmonton, AB Canada; 18 Allied Health, Sydney Local Health District, Sydney, Australia; 19 Department of Physical Therapy, University of British Columbia, Vancouver, BC Canada; 20 Centre for Heart Lung Innovation, University of British Columbia, Vancouver, BC Canada; 21 St. Joseph’s Healthcare Hamilton, Hamilton, ON Canada

**Keywords:** Chronic obstructive pulmonary disease (COPD), Pulmonary rehabilitation, Balance training, Randomized controlled trial, Project management

## Abstract

**Background:**

Pulmonary rehabilitation (PR) is accepted as standard care for individuals with COPD. We conducted an international, multi-centred randomized controlled trial (RCT) to determine if adding balance training to PR would reduce the incidence of falls in people with COPD. While there have been many trials investigating the effectiveness of PR, few have involved international collaboration. Successful execution of rehabilitation trials requires a significant investment of time, staffing, and resources. With the recent completion of the Balance Training for Fall Reduction in COPD RCT, we report on the design, implementation, and execution of our trial using project management phases. We also highlight our lessons learned for consideration in future multi-centre rehabilitation trials.

**Methods:**

This was a retrospective review of the planning, preparation, timelines, and personnel training involved in the execution of this study using four of the five project management phases described by Farrell et al. in 2010: (1) initiation, (2) planning, (3) execution, and (4) monitoring and controlling. We report descriptive statistics as percentages and counts and summarize our lessons learned.

**Results:**

Ten outpatient PR programs in three continents participated. Thirty-one personnel worked on the trial across all sites. Enrolment began in January 2017 and was suspended in March 2020 due to the COVID-19 pandemic. Approximately 1275 patients were screened, 455 (36%) were eligible, 258 (57%) consented, 243 (53%) participated, and 130 (61%) completed the 12-month follow-up assessment. Lessons learned through our experience included (1) ensuring awareness of funder policies and considering the impact on collaborating sites; (2) preparing for the possibility of human resource and program disruptions; (3) anticipating site dropout and having a contingency plan in place; (4) planning and monitoring process measure data before, during, and after trial initiation; (5) ensuring frequent and consistent communication with and between collaborating sites; (6) maximizing features of database platform to ensure data set completeness and controlled data access; and (7) identifying strategies for increasing patient engagement in a high-demand study.

**Conclusions:**

We identify seven lessons learned through our experience conducting an international, multicentre rehabilitation-based RCT. These lessons can provide guidance to other trialists conducting studies with similar logistics and may assist with future trial planning and implementation.

**Supplementary Information:**

The online version contains supplementary material available at 10.1186/s13063-024-08251-1.

## Background

Chronic obstructive pulmonary disease (COPD) is a leading cause of morbidity and mortality, and, with an ageing worldwide population, the prevalence of COPD is expected to increase in the coming years [1]. While much of COPD management focuses on changes within the respiratory system, secondary impairments in skeletal muscle strength and endurance, exercise capacity, and functional mobility are also present with this population [2, 3]. There is now recognition that people with COPD are at an increased risk of falls, which are associated with increased morbidity and mortality [4–6]. People with COPD are reported to have a higher prevalence of falls as compared to the general elderly population [5, 7]. Compared to controls, people with COPD have higher levels of balance impairments, with deficits in three specific balance subsystems: biomechanics (i.e. strength, range of motion, posture), transitions (i.e. changes in body positions), and gait (i.e. stability during ambulation) [8]. In addition to physical impairments, people with COPD often have concomitant comorbidities that can further increase the risk of falls, including polypharmacy, cognitive impairment, malnutrition, and various other health conditions including osteoarthritis and osteoporosis [5]. Pulmonary rehabilitation (PR) is recommended as standard management for people with COPD as there is evidence to support its efficacy in improving symptoms, exercise tolerance, and health-related quality of life. Nevertheless, international PR guidelines do not consistently include fall prevention recommendations or balance training to reduce falls [9–11].

Successful implementation of an RCT requires significant human, logistical, and financial resources throughout the course of a trial’s planning, preparation, and execution. Unfortunately, many RCTs still fail to meet a priori targets, including adequate participant enrollment, appropriate data collection, and meeting budgetary constraints [12, 13]. In a 2007 analysis of 114 multi-centre intervention trials, 45% failed to reach 80% of their target sample size [14]. Nearly one third of these studies also required an extension in both time and resources to complete the trials successfully [14]. A subsequent 2013 review of 73 trials noted similar findings; only 55% of trials met their recruitment targets while 45% required an extension of their timeline in order to obtain adequate enrollment [15].

Given the challenges researchers face when planning and executing an RCT, a project management approach has been suggested as a means of identifying potential barriers, adequately managing human and financial resources, and monitoring progress towards a priori objectives [12, 13]. Farrell and colleagues suggested the use of the five project management phases as a means of organizing and monitoring trial conduct [12]. These five processes include:

1. Initiation: encompasses project definition, determining research feasibility, and obtaining authorization to begin [12, 13, 16].
2. Planning: requires establishing study objectives, scope of research, and delineation of roles and responsibilities of study personnel [12, 16].
3. Execution: involves the allocation of study resources and support to the research team in order to complete study aims [13].
4. Monitoring and controlling: occurs in tandem with execution. This phase includes measuring study progress, identifying barriers to success, and modifying the project as needed to remain on track [13].
5. Analysis and reporting: involves the formal completion of the study, including collating data and dissemination of results [13].

The core trial team conducted a single-centre RCT to examine the impacts of adding a balance training program to PR compared to PR alone [17]. Large and clinically important differences were found in balance performance in the intervention group as compared to the control group. Subsequently, we initiated an international, multi-centre RCT in four countries across three continents to confirm these findings and determine the impact of the intervention on the incidence of falls (NCT02995681). This international trial differed significantly from the single-centre study in terms of expected sample size (39 participants in the single-centre study versus a target of 400 in the international study), number of sites (one PR site versus 10), and study duration (6 to 8 weeks versus 12 months). Therefore, the objective of this paper is to retrospectively review the planning, preparation, timelines, personnel training, and additional resources required in the execution of the larger RCT and to summarize lessons learned using the first four project management phases as described by Farrell et al. in 2010 [12].

## Methods

### Overview of RCT of Balance Training for Fall Reduction in COPD

We conducted an assessor-blinded, international multi-centre RCT evaluating the long-term effects of tailored balance training on the rate of falls in individuals with COPD enrolled in a PR program. Ten outpatient PR centres across four countries were involved in this RCT (Table 1). Individuals were eligible for inclusion if they had (1) a diagnosis of COPD based on the Global Initiative for Chronic Obstructive Lung Disease (GOLD) criteria; (2) a self-reported decline in balance, a fall in the last two years or a recent near fall; and (3) the ability to provide written informed consent [18]. Participants were randomized to the intervention group who received 30 min of tailored balance training three times per week (two in-person sessions, one independent session) plus standard PR versus the control group who were assigned to standard PR only. The sample size target was 400 participants across the 10 centres. The primary outcome measure was the incidence of falls at 12-month follow-up. Secondary outcomes were the Berg Balance Scale (BBS), the Balance Evaluation Systems Test (BESTest), the Activities-Specific Balance Confidence (ABC) scale, and the 30-s repeated chair stand test. Outcome measures were obtained by blinded assessors at baseline, end of PR, and at a 12-month follow-up. We set an a priori threshold for PR and balance training completion of 70%. Further details of our protocol are available in a previous publication [18].

**Table 1:** Site locations

Site name	City, state/province	Country
South Tees Hospital, NHS Foundation Trust	Middlesbrough	England, UK
North Tees and Hartlepool Hospital, NHS Foundation Trust	Middlesbrough	England, UK
University of Aveiro	Aveiro	Portugal
Alfred Health	Melbourne	Australia
Royal Prince Alfred Hospital	Camperdown, New South Wales	Australia
Western Health	St. Albans, Victoria	Australia
Colchester East Hants Health Centre	Truro, Nova Scotia	Canada
G.F. MacDonald Centre for Lung Health	Edmonton, Alberta	Canada
St. Paul’s Hospital	Vancouver, British Columbia	Canada
West Park Healthcare Centre	Toronto, Ontario	Canada

*NHS* National Health Service

### Balance in pulmonary rehabilitation management team

All trial-related activities were organized and directed through a core trial team at the lead site (West Park Healthcare Centre, Toronto, Ontario, Canada). This core trial team included the primary investigators (M.B., R.G., D.B.), a full-time (37.5 h/week) physiotherapist research coordinator (RC) (C.E.), a statistician, and an economic analyst. Each collaborating site had one or two identified site leads who were responsible for disseminating the study protocol to local study staff and overseeing all local study operations. Other site study personnel included balance trainers and blinded outcome assessors, both of whom were physiotherapists.

The central RC maintained bi-monthly contact via emails with the site leads. These emails consisted of study updates, responding to protocol questions from site leads, study enrollment status, and identified barriers to protocol implementation with suggested strategies to overcome them. A data monitoring committee was also established prior to the commencement of the study to monitor for adverse events during the study. Four of ten sites required the local site lead to perform at least one study-related procedure, including participant screening, obtaining consent, balance training, outcome assessment, and data entry. Seven of the ten sites employed RCs to assist with these activities, with funding for the RCs supported by participant enrolment.

### Project management overview

We performed a retrospective review of all trial activities, timelines, study milestones, communications, and the roles and responsibilities of trial personnel involved in the balance RCT. We examined all data sources from study preparation to completion, including grant applications, email correspondence between various team members, the previously published study protocol, research ethics board (REB) applications and responses, screening logs, expense reports, and database entries. After reviewing the available data sources, further information was obtained from all site leads via an electronic survey. This information included the type and length of local personnel training, turnover of study personnel, responsibilities of site leads, and adequacy of the study budget and central RC communication.

This manuscript was guided by the project management phases as outlined by Farrell et al. in 2010 [12]. We have also considered previous project management literature to assist with organizing trial activities into each phase, in particular the 2019 paper by McCaskell et al. as it reviewed the stages and lessons learned while conducting a multi-centre rehabilitation trial [16].

### Statistical analysis

We report descriptive statistics as percentages and counts for binary data and medians and interquartile ranges for continuous data. The lessons learned are summarized using the project management phases of Farrell et al. [12].

## Results

### Initiation

The initiation phase included both trial-level activities (i.e. establishment of a study team, development of the study protocol, applications for funding) and site-level activities (i.e. research ethics board (REB) approvals).

The lead investigators invited other national and international researchers in the field of pulmonary rehabilitation to collaborate on the project. Identification of potential co-investigators was informed by prior academic research collaboration or their known experience in conducting research in individuals with COPD or in pulmonary rehabilitation. The resultant study team was an experienced research group with 90% having conducted single-centre RCTs, 85% having conducted single-country multi-centre RCTs and 30% having conducted international, multi-centre RCTs.

Development of the study protocol was informed by our prior experience with a single-site RCT on balance training in PR [17] along with the input of all named co-investigators. Multiple rounds of editing refined the protocol prior to submission to the target funding agency. The lead investigators (M.B., R.G., D.B.) along with named co-investigators (A.H., A.L, A.M., E.S., G.D., J.A, L.S, M.K.S., P.C., R.M., S.L.H., X.F.) submitted three independent Canadian Institute for Health Research (CIHR) grant applications (one operating grant and two project grants) between 2015 and 2016 application periods. We were awarded the 2016 CIHR Operating Grant (application success rate:1/3 = 33%), which provided funding for 3 years. The grant was used to fund study personnel wages, equipment acquisition, outcome measure licence fees, and other study operational costs.

Once funding was secured, we proceeded with REB applications, prioritizing applications to the lead site (West Park Healthcare Centre, Toronto, Canada) and the academic institution responsible for holding and administering the CIHR grant (University of Toronto, Toronto, Canada). We received REB approval from West Park Healthcare Centre on November 29, 2016, and the University of Toronto on January 20, 2017. Registration of the study with ClinicalTrials.gov was completed on December 16, 2016 (NCT02995681). Once REB approval was obtained from West Park Healthcare Centre and the University of Toronto, REB applications for the collaborating sites were worked on concurrently. All collaborating sites received local REB approval within the first seven months of 2017, with the final approval being granted on July 25, 2017, for the University of British Columbia, Canada. We subsequently published our protocol in 2017 [18].

### Planning

We undertook further site-level activities during the planning phase. Sub-grant and data-sharing agreements were drafted when REB approval was obtained with each collaborating site The sub-grant agreements were legally binding agreements between participating institutions (i.e. CIHR main grant holder and collaborating sites) that outlined the responsibilities of the collaborating site, the funding allocation for the collaborating site and the funding term. Data sharing agreements reflected institutional requirements to ensure data security when collaborating sites are providing data to the lead site. The initial subgrant term was July 1, 2016, through to June 30, 2019. Executing subgrant agreements posed significant challenges both in terms of the limitations imposed by funder policies and in managing institutional expectations, communication, and timelines. CIHR does not permit international collaborating sites or non-CIHR-approved Canadian sites (West Park Healthcare Centre) to hold (i.e. self-administer) subgrant funds. As a result, these sites were required to invoice the University of Toronto for equipment, incidental, and personnel costs after they were incurred. To mitigate the financial burden this imposed during the study planning phase, the central RC ordered the necessary balance assessment/training equipment on behalf of the non-CIHR-approved sites.

Collaborating site subgrants were determined based on an estimate of the personnel costs related to moving a participant through the complete study protocol. The balance trainer was allotted 20.5 h per participant enrolled for tailored balance training sessions, development of home program prescription, monthly phone calls and home visits. The outcome assessor was allotted 9.5 h per participant enrolled for recruitment, data collection from the medical records, outcome measure assessments, and data entry. The University of Aveiro (Portugal) subgrant provided funding for a full-time physiotherapist for two years to offer a PR program locally, therefore providing a study recruitment opportunity. All sites received $500.00 CAD for equipment and $1500.00 CAD for other study operational costs, such as participant parking fees and study package development including postage for monthly fall diary calendars.

Training material and the study manual (study protocol, CRFs, balance training flowsheets and outcome measures) were prepared by the central RC and provided to site leads via email. Site leads were not provided with formal study protocol training as they were instrumental in the protocol development. If clarification of any information was required, video teleconferences and phone calls were scheduled with the central RC.

Each site was then responsible for training local study personnel. The exception was Australia, where one site lead (Alfred Health) provided on-site training for all Australian sites (Alfred Health, Royal Prince Alfred Hospital and Western Health). This was a local decision intended to reduce the burden of training across the individual sites. Training of outcome assessors and balance trainers was provided by RCs at three sites, a clinician researcher at one site, and by the site leads at six sites. Most sites provided in-person, practical training for their study personnel, with one site using a combination of didactic/online videos and in-person training. On average, most training sessions were one to two hours in length for both outcome assessors and balance trainers. Several centres noted that minimal training of outcome assessors and balance trainers was required to achieve competence on the clinical balance measures and the balance training intervention given their background as physiotherapists who had experience in balance assessment and training. In total, 14 outcome assessors and 17 balance trainers were trained across all ten sites. As nearly all site leads were physiotherapists, they were able to provide coverage for outcome assessors due to illness or vacation to ensure continuity with study protocols. At least two sites acknowledged the use of their site leads to backfill for research personnel. While this was a practical solution to staffing shortages, there were implications to site funding given funder policies.

An online database (REDCap®) mirroring the hard-copy data collection forms was created by the central RC during this phase. This allowed for seamless data sharing between the collaborating sites and the lead site.

### Execution

The execution phase included both patient-level (i.e. screening and enrolment, balance training, outcome measure assessment) and site-level (i.e. staffing, data entry and cleaning, ethics amendments and annual renewals, funding, subgrant agreements) activities.

#### Screening and enrolment

Study enrolment began in January 2017 and was suspended in March 2020 at the onset of the COVID-19 pandemic. Enrolment resumed at one site between January 2021 and May 2021 however it was not sustained due to the ongoing effects of the pandemic. We screened a total of 1275 patients across the ten sites over the 38.5-month study period: 458 (36%) were eligible, 258 (56%) consented to participate, and 243 (54%) participated in the study. A mean of 6.4 participants per month were enrolled across all sites.

Screening for participants meeting the eligibility criteria was performed by a variety of research and clinical personnel across all sites. This was most often conducted by clinical staff (7/10 sites), followed by balance trainers (3/10 sites), site leads (2/10 sites) and both RCs (1/10 sites) and assistants (1/10 sites). Informed consent was also a shared responsibility between several roles, including clinical staff (3/10 sites), outcome assessors (3/10 sites), site leads (2/10 sites), research assistants (2/10 sites), and RC (1/10 sites).

Randomization was performed using a centralized randomization process. The central RC created a block randomization table using a free online random number generator (https://www.randomizer.org/) and sequential sealed envelopes were then created for each site. The local outcome assessor or site lead notified the central RC when the baseline assessment of a consenting participant had been completed. The central RC sequentially opened a sealed envelope and advised the balance trainer or local RC/site lead of the participant’s group allocation. All sites found this means of randomization effective.

#### Balance training

Of the 31 additional research personnel trained for the study, 16 (53%) provided balance training to study participants across our ten sites. Three site leads and the central RC in Toronto also provided balance training including covering for research staff illness and vacation. The attendance rate for balance training sessions during PR among participants in the intervention group was 80%. Reasons reported for missed balance training sessions included: acute exacerbation of COPD (AECOPD), co-morbidity flare-up, conflicting appointments, transportation challenges, and staffing limitations (including planned vacations, unexpected illness, research, and clinical staff vacancies).

#### Outcome measure assessment

Fourteen study personnel (47%) performed outcome assessments across all sites. Three site leads also conducted assessments as needed. Each site determined their own method to notify the outcome assessors of a pending assessment. Email, phone calls, text messages, and in-person communication were the most common strategies used. All outcome assessors were blinded (including site leads if they filled this role), and no adverse events occurred during the performance of any of the secondary outcome measures.

Of the 258 individuals who consented to participate in the study, 243 (94%) completed the baseline assessment prior to being randomized. Three additional participants were erroneously randomized prior to completion of the baseline assessment however they did not participate in the trial. One hundred and seventy-five participants (68%) completed at least part of the post-PR assessment (complete dataset = 169, partial due to COVID-19 = 6) and 130 (50%) completed at least part of the 12-month post-PR follow-up assessment (complete dataset = 121, partial due to COVID-19 = 9). Among the 175 participants who completed at least part of the post-PR assessment, we received 76% of their monthly fall diaries and exercise calendars. Similarly, among the 130 participants who completed at least part of the 12-months post-PR assessment, the average return rate for the monthly fall and exercise diary calendars was 80%.

We utilized an intention-to-treat analysis and attempted to collect outcome measure data for all participants, including those who did not meet our a priori completion threshold for PR and/or balance training. Of the 80 participants (33%) who did not meet our a priori completion threshold for PR and/or balance training, we were able to collect post-PR assessment data from 30 participants (46%) and 12-month follow-up assessment data from 17 participants (21%). An additional 7 participants (9%) only submitted monthly falls diary calendars for the 12-month follow-up period but did not participate in either the post-PR assessment or the 12-month follow-up assessment.

#### Staffing

In addition to the lead PI’s and the site leads, 31 study personnel were involved in the trial (Table 2).

**Table 2:** Staffing complements/turnover at sites

Site name	Outcome measure assessors (*n*)	Balance trainers (*n*)	Turnover (Y/N)
South Tees Hospital, NHS Foundation Trust	1	1	N
North Tees and Hartlepool Hospital, NHS Foundation Trust	1	1	N
University of Aveiro	1	1	N
Alfred Health	1	4	Y (BT only)
Royal Prince Alfred Hospital	2	1	Y (OCM only)
Western Health	1	1	Y
Colchester East Hants Health Centre	Site lead only	1	N
G.F. MacDonald Centre for Lung Health	2	2	Y (OCM and BT)
St. Paul’s Hospital	1	1	N
West Park Healthcare Centre	4	4	Y

*Abbreviations: OCM* outcome measure assessor, *BT* balance trainer, *Y* yes, *N* no

#### Data entry and cleaning

Paper-based data collection forms were utilized, and data was then entered into the REDCap® online database. Each site was responsible for their own data entry. The core trial team could view data from all sites while individual sites could only view their own data. Each site lead was responsible for reviewing and cleaning local study data. The central RC performed periodic data checks and followed up with emails to sites missing data or those with obvious data entry errors. To maintain blinding of the outcome assessors who were responsible for entering assessment data into REDCap®, group allocation and balance training data were not entered into the database but were kept secure at each site on the hard copy CRFs and balance training flowsheets. This data was added to the complete study database when all participants completed the protocol. Final data cleaning was completed in May 2022.

#### Ethics amendments and annual renewals

We submitted seven REB amendments across five participating sites (University of Alberta (*n* = 2), LaTrobe University (*n* = 1), University of British Columbia (*n* = 1), Teesside University (*n* = 2), and Nova Scotia Health Authority (*n* = 1)). Amendments were required for a variety of reasons including the following: (1) change of study staff; (2) clarification of protocol details including submission of additional data collection forms, newly drafted script for monthly phone calls and the addition of a digital database (REDCap); and (3) change in recruiting site.

Annual REB renewals were managed by site leads in the UK, Australia, Portugal, Alberta (Canada) and British Columbia (Canada), while the central RC was responsible for REB renewals for West Park Healthcare Centre, the University of Toronto, and Nova Scotia Health Authority (Dalhousie University) (Canada).

#### Funding

Reimbursement for personnel costs posed a significant challenge for international and non-CIHR-approved Canadian sites throughout the study. Invoicing for services already rendered, institutional processes for drafting invoices, institutional policies for receiving invoices, along with the impact of international currency exchange and invoice payment terms all contributed to significant delays (45–90 days) in allocated subgrant funds reaching collaborating sites.

Given slower than expected enrolment and the effects of the COVID-19 pandemic, we requested and were granted two 1-year no-cost extensions (March 3, 2020; May 5, 2022) and CIHR further provided an automatic one-year no-cost extension (June 3, 2020). As a result, the term of the subgrants was extended to March 31, 2024.

#### Subgrants

Twenty subgrant amendments were required across six of our participating sites (West Park Healthcare Centre (*n* = 5), University of Alberta (*n* = 4), LaTrobe University (*n* = 3), Monash University (*n* = 2), Teesside University (*n* = 3) and University of Sydney (*n* = 3)). Amendments were executed for a variety of reasons including (1) extension of subgrant terms to reflect the three time-only extensions from CIHR; (2) increase in subgrant funding to support extension of lead RC position to match study timelines; (3) update the statement of work to reflect increased enrolment targets at select sites; (4) increase funds allocated under the subgrant due to increased enrolment targets; and (5) change in academic affiliation of site leads.

### Monitoring and controlling

This phase occurred simultaneously with execution and included both site-level and trial-level activities.

#### Screening and enrolment

The core trial team kept a record of screening and enrolment targets throughout the course of the study. Our initial target sample size was 400 participants. The withdrawal of two recruiting centres (St. Paul’s Hospital, British Columbia and Western Health, Australia) due to difficulties with recruitment and staffing limitations made meeting the required sample challenging. As a result, the remaining eight sites were asked to increase their enrollment targets and the study timeline was extended beyond December 2019. The G.F MacDonald Centre for Lung Health (Alberta, Canada) agreed to increase enrolment by 20 participants, Alfred Health (Australia) by 10, and Royal Prince Alfred Hospital (Australia) by 10. North Tees and Hartlepool Hospital (United Kingdom) was added as a recruitment site in response to the withdrawal of the Canadian and Australian sites, with an enrolment target of 15 participants. Unfortunately, the COVID-19 pandemic interrupted study progress in March 2020, leading to suspension of recruitment. While one site (Royal Prince Alfred Hospital, Australia) was able to resume recruitment for a brief period in 2021, no other site resumed enrollment. We closed the trial with 243 participants enrolled. Based on an enrolment rate of 6.4 participants per month across all sites, we estimate that it would have taken another 24 months to meet our minimum sample size requirement.

Several factors influenced our decision to end the trial early. First, the prolonged break in study operations because of the COVID-19 pandemic resulted in study staff seeking alternative employment, leaving limited research personnel to resume study operations. Second, in-person PR enrolment remained limited in the later stages of the pandemic, and this would have resulted in poor study recruitment. Finally, while most PR programs pivoted to virtual care delivery at the start of the pandemic and continued with this care delivery model, online balance assessment and training were not deemed feasible for study participants due to safety concerns.

#### Balance training

Among participants who remained engaged in the study and attended the post-PR assessment session, the average attendance was 12 of the expected 16 balance training sessions (75%). Balance training sessions were missed for a variety of reasons, including participants not attending PR, AECOPD, co-morbidity flare-ups, participant fatigue, appointment conflicts, limitations in transportation, and staff shortages. Given the nature of the pandemic and the elevated risks associated with the patient population, in-person outpatient PR programs and non-essential research activities were suspended in March 2020. Some collaborating sites pivoted to virtual delivery of PR however, in consultation with lead PI’s and local clinical staff, it was deemed unsafe to assess balance and deliver the balance training intervention through an online platform.

#### Outcome measure assessment

Several issues arose concerning outcome measure assessments. Two collaborating sites did not initiate the fall and exercise diary calendars until participants completed PR and some participants were booked for the final outcome assessment session 12 months from enrolment in the study instead of 12 months post-completion of PR (+ / − balance training intervention). Once these issues were identified, the central RC clarified the protocol timelines, encouraged more timely data entry into the shared database, and instituted more frequent data checks.

Overall, some outcome measure assessments were missed due to patient availability, declined appointments, AECOPD, and worsening co-morbid conditions. The pandemic also prevented in-person assessment, therefore limiting outcome measure assessment to the return of monthly fall and exercise diary calendars, monthly phone calls for healthcare utilization and loss of productivity data and completion of questionnaires at post-PR and 12-months post-PR follow-up time frames.

#### Data entry and cleaning

Data entry remained the responsibility of each site with the central RC reviewing all data and following up with sites with any data concerns or questions. Typical reasons for missing data included participant failure to return monthly fall and exercise diary calendars, staff being unable to reach participants by phone for monthly healthcare utilization and loss of productivity data collection, and staff being unable to book participants for outcome assessment sessions (unable to reach, participant declining, participant illness, COVID-19 pandemic restrictions).

### Lessons learned

Our experience designing and implementing a large, multi-site, international randomized trial provided multiple opportunities to reflect on lessons learned which may help future trialists planning international rehabilitation trials. We identified seven key lessons learned including the importance of (1) ensuring awareness of funder policies and considering the impact on collaborating sites; (2) preparing for the possibility of human resource and program disruptions; (3) anticipating site dropout and having a contingency plan in place; (4) planning and monitoring process measure data before, during, and after trial initiation; (5) ensuring frequent and consistent communication with and between collaborating sites; (6) maximizing features of the database platform to enhance data-set completeness and control data access; and (7) identifying strategies for increasing patient engagement in a high-demand study. These lessons are organized by trial management phase in Table 3.

**Table 3:** Seven lessons learned from the balance in PR RCT

Lessons learned	Project management phase(s)	Suggestions for future rehabilitation trials
Ensuring awareness of funder policies and considering the impact on collaborating sites	Initiation Planning	- Consider funder guidelines when compensation planning with collaborating sites - Consider securing local funding to help offset funding delays at collaborating sites
Preparing for the possibility of human resource and program disruptions	Initiation	- Consider background of site leads when planning for possible human resource disruption - Thorough evaluation of program volume and experience with research trials to establish a realistic enrolment plan - Consider potential for disruptive local/global events and have contingency plan for study continuity
Anticipating site dropout and having a contingency plan in place	Planning	- Contingency plan for additional sites to join - Prepare participating sites for potential increased enrolment - Plan budget for potential increased timeline to complete study
Planning and monitoring process measure data before, during, and after trial initiation	Planning Execution Monitoring and Controlling	- Proactively identify and collect process data in database - Create and update training and screening logs - Accurately measure time to train and start study to inform future trials, and budgeting
Ensuring frequent and consistent communication with and between collaborating sites	Planning Execution	- Consider and budget for onsite visits from lead site personnel to assist with protocol initiation - Identify appropriate subleadership roles at participating sites
Maximizing features of the database platform to enhance data set completeness and control data access	Planning Execution Monitoring and Controlling	- Improve ease of access to all data and avoids need for scanning/faxing/mailing paper forms - Minimize risk of unblinding assessors
Identifying strategies for increasing patient engagement in a high-demand study	Planning Execution Monitoring and Controlling	- Offer hybrid models of training/assessment if appropriate for study population and intervention - Limit redundancy in outcome measures to reduce impact on participants - Ensure accurate funding to cover all incidental costs of study participation - Consider remuneration for protracted study involvement

## Discussion

Randomized controlled trials in rehabilitation are particularly complex and their success is dependent on multiple variables. The RCT of Balance Training for Fall Reduction in COPD was the first large-scale, international, multi-centre RCT to evaluate the impact of a tailored balance training program on fall reduction in people with COPD. In addition to the lead PI’s and the named co-investigators, 31 study personnel were trained across four countries and 130 participants completed at least a portion of the 12-month follow-up assessment prior to early study termination due to the COVID-19 pandemic. Unfortunately, we did not meet our sample size targets and identified issues with both participant recruitment and retention throughout the study. The use of project management principles can provide structure and organization to ensure that study milestones are accomplished efficiently and accurately, and to help install safeguards to mitigate potential barriers that could impact the relevancy of study findings. We gained valuable experience with international trial management and this report summarizes our lessons learned [12].

Intervention fidelity, defined as the extent to which an intervention was delivered as planned, is an important factor to consider when trying to determine if a lack of intervention efficacy is the result of intervention failure versus implementation failure [19, 20]. Rehabilitation studies often involve complex and multimodal interventions with many opportunities for reduced protocol fidelity, including staffing challenges, lack of consistent personnel training, and divergent treatment execution. Frequent and consistent communication across sites has been identified as a key factor in successful trial management [13, 16]. While all site leads for the RCT of Balance Training for Fall Reduction in COPD were familiar with the study protocol, provision of the study manual may not have been sufficient to support protocol implementation. Furthermore, each of the ten sites were responsible for training their study personnel. Each site used a differing combination of didactic and virtual training for balance assessment and training, and this may have resulted in differing outcome measure assessment and balance training. We encourage future trialists to consider in-person training at collaborating sites for both physical outcome measures and treatment interventions to increase the likelihood of protocol fidelity and consistency between sites. As this option may not be financially feasible depending on study funding and geographic locations, another strategy is for collaborating sites to video record study personnel conducting trial activities, which would then be reviewed and critiqued by the trial coordinators. In our trial, this strategy would have been appropriate both for the Balance Evaluation Systems Test (BESTest) and for the tailored balance training protocol. Another tactic to ensure consistency between sites involves increasing the frequency of communication between the lead and collaborating sites after a site’s first participant enrollment. We believe this could be an opportunity to review protocol milestones and provide proactive feedback around protocol fidelity as opposed to retroactively identifying protocol deviations.

Previous rehabilitation trials have identified dependency on frontline healthcare providers to carry out study interventions [16]. In their pilot RCT of in-bed cycling, Kho and colleagues noted that staffing shortages limited the ability to enrol qualifying participants into their study. A total of 31 recruitment weeks across five sites were lost due to staffing shortages from physiotherapist turnover, leaves of absences, and vacations [21]. These findings were echoed in the retrospective review of the A Very Early Rehabilitation Trial for Stroke (AVERT) trial, which reported a 10% loss in time to recruit their target sample due to personnel leaves of absence [22]. Nine of our 10 site leads were physiotherapists and the majority of site leads acknowledged participating in at least one trial activity such as participant screening and balance training. Several site leads provided coverage for outcome assessor absences which prevented study protocol violations and also assisted in maintaining blinding of group allocation as other study personnel did not have to conduct these assessments. Future rehabilitation trialists should consider identifying site leads of the appropriate discipline as a contingency plan for staffing shortages or short-term leaves of absence. However, funder policies must be considered when employing this strategy as named grant applicants may not be eligible for compensation related to study activities.

Pilot studies play an integral role in evaluating the likelihood of procedural success of a large-scale trial [23]. They provide important information about the feasibility of recruitment and retention strategies, protocol implementation, and resource utilization [24]. Given the inherent complexity of rehabilitation interventions, which often require significant human resources and a high demand of enrolled participants, pilot studies are necessary to understand facilitators and barriers to future trial success [23, 24]. Our large-scale multi-centre trial was informed by a 2013 single-centre RCT in which the addition of a balance training program to usual PR led to statistically significant short-term improvements in clinical balance measures in the intervention group as compared to the control group [17, 23, 24]. Despite undertaking this rigorous single-centre RCT, our trial still encountered challenges that impeded its success. Lessons learned conducting a shorter duration single-centre RCT versus managing a long-term international multi-centre trial highlight the importance of evaluating as many aspects of trial implementation as possible in the pilot phase. For example, funder policies were not impactful in the single-centre RCT and therefore did not inform our planning for the international multi-centre trial. We also identified recruitment and retention challenges over 12 months that were not noted in the pilot study which was conducted over a shorter duration (6–8 weeks) in an inpatient environment. We recommend that pilot studies be conducted in the same care environment (i.e. outpatient versus inpatient) and include a minimum of two sites in different regions as a means of assessing implementation success from various perspectives.

Reimbursement for personnel costs remained a significant challenge throughout the entirety of trial proceedings. The need to invoice for services already rendered and multi-layered processes at both the payee and payor institutions resulted in significant delays in collaborating sites receiving funds. As a result of this funding model, sites reported difficulty with both hiring and retaining study personnel. The success of any rehabilitation trial is dependent upon the involvement of allied health personnel. Recruitment challenges and high staff turnover will negatively impact study function, lead to timeline delays, and likely impact the study budget. Our team recommends ensuring all potential collaborating sites are aware of funder policies before committing to study participation. We further encourage trialists to apply for local start-up funding when the policies of the primary funder limit who can hold and self-manage subgrant funds.

The COVID-19 pandemic created a significant challenge for many rehabilitation trials around the world and our trial was unprepared for such a disruption. While some rehabilitation trials were able to pivot and continue with participant enrolment after a delay [25], we were unable to successfully resume study activities. This disruption led us to complete the study with enrolment of 258 of the targeted 400 participants. In contrast, other rehabilitation trials were able to implement mitigation strategies to minimize missing data from study participants during the pandemic. For example, in the international CYCLE RCT[25], strategies such as prioritization of the primary outcome measure for enrolled participants meant they were eventually able to resume enrolment at 10 of their 15 international sites. The authors recommended the use of the CONSERVE framework as a means of documenting necessary protocol deviations in the case of an unforeseen disruption [26]. Given the impact of the COVID-19 pandemic on in-person research operations, future trialists should have a continency plan for mitigating data loss, preserving patient enrolment, and preparing for full resumption of study activities when the situation permits.

Prior to the onset of the COVID-19 pandemic, two collaborating sites had to withdraw from study participation due to staffing challenges. To meet our target sample, we requested that the other collaborating sites increase their enrollment targets. Four collaborating sites who were having enrolment success (G.F. MacDonald, South Tees, Alfred Health, Royal Prince Alfred) were able to accommodate this request however it likely put undue pressure on them as they were nearing their original enrolment targets. Having a contingency plan of additional collaborating sites who might be willing to join mid-study is an important consideration to maintain timelines and achieve enrollment targets.

While rehabilitation protocols can be complex for study personnel to implement and sustain, researchers need to consider the impact of high-demand interventions on study participants. Identifying strategies to increase participant engagement is an essential task and one that we recommend initiating at the study onset. Suggestions include appropriately compensating participants for their time and for study participation costs, such as parking and transportation. While parking costs were covered for trial-specific, in-person outcome measure assessments, we did not provide parking reimbursement for PR sessions. This additional gesture of appreciation may have impacted participant attendance and retention and is worth considering for inclusion in study budgets for future trialists.

Our study had challenges with participant retention given the significant engagement required of participants (i.e. three outcome measure assessments, monthly fall and exercise diary calendars, monthly phone calls for healthcare utilization data collection and home program progression and three home visits) during the 12-month follow-up period. Where possible, trialists may consider a hybrid model of in-person and virtual engagement within their study protocol. For example, virtual assessments or interventions may reduce participant burden by eliminating travel time and cost and this may assist with participant enrolment and retention. Our challenges with retention were further exacerbated by the pandemic given that all actively enrolled participants ceased to have access to PR, or the in-person balance training intervention and in-person outcome measure assessment sessions were also not feasible. Unfortunately, we felt we could not offer virtual balance assessment or training sessions due to concerns regarding participant safety with respect to risk of falls as well as concerns about fidelity to the original intervention. Notably, our intervention was designed as an adjunct to standard PR and in-person PR was not running in any of the study jurisdictions. Studies could consider offering virtual outcome assessments or rehabilitation interventions as a means of minimizing the burden on participants if participant safety issues are not a concern.

There is limited literature on the use of a project management framework in rehabilitation research. Two previous studies have applied a project management lens to review their trial processes and procedures. Arundel and Gellatly applied a project management framework to the Obsessive Compulsive Treatment Efficacy Trial (OCTET), a multi-site RCT aimed at evaluating the effectiveness of two low-intensity outpatient interventions for adults with obsessive–compulsive disorder awaiting cognitive behavioural therapy [13]. McCaskell and colleagues retrospectively analyzed study activities, milestones, and timelines of a pilot RCT implementing a novel rehabilitation intervention with a critically ill patient population [16]. Both authors highlighted open communication between study personnel and the research team, consistent support of study personnel by research staff, the provision of clear and detailed study resources, and tailored face-to-face training as important factors that contributed to the study’s success [13, 16].

Our analysis has limitations. First, our process data was collected and interpreted retrospectively. We did not structure our data collection with these outcomes in mind and as such there are gaps in our process management outcomes. Some data were not captured and therefore cannot be accurately reported. We surveyed the site leads to fill some gaps in our knowledge and this may have led to misrepresentation of some trial processes as they were required to recollect activities from years prior. We did not survey the 31 additional study personnel who performed study activities and as such, our results may not accurately reflect their experiences or opinions of trial function and conduct. Second, our results may not be generalizable to all types of rehabilitation populations and sites. For example, our study participants were individuals with COPD in an outpatient environment and our findings may not be appropriate for trialists conducting studies with individuals with different diagnoses in an inpatient environment or at sites with differing program structures and staffing complements. Finally, we acknowledge that our suggestions for future trialists are potentially costly and may impact study budgets. However, we believe these suggestions can help mitigate some of the challenges we faced, and they may contribute to improved trial outcomes.

This work also has several strengths. First, we are contributing to the sparse field of literature reviewing an international rehabilitation trial using a project management lens. Our analysis covers all stages of study development and implementation and identifies lessons learned from four project management phases which may assist future trialists. Second, it builds upon previous literature and adds a novel perspective surrounding the conduct of an international multi-centre trial with a complex outpatient population. Third, insights are based on the experience of coordinating many study personnel across multiple countries and the lessons learned were garnered from researchers and physiotherapists with years of PR and academic research experience. Finally, the lessons learned are relevant and important considerations for all researchers designing rehabilitation trials, both single and multicentre.

## Conclusions

Randomized controlled trials in rehabilitation are complex and their success is dependent upon thoughtful methodological design as well as skillful coordination of multiple processes, personnel, and resources. Standardized process management provides researchers with a structured method of developing, conducting, and evaluating an RCT which can improve the likelihood of a successful study. We recommend that future RCTs of rehabilitation adopt an established project management framework.

### Supplementary Information

Supplementary Material 1.

## Data Availability

The datasets used and/or analysed during the current study are available from the corresponding author on reasonable request and ethics approval.

## Abbreviations

AECOPD: Acute exacerbation of chronic obstructive pulmonary disease
BESTest: Balance Evaluation Systems Test
BT: Balance trainer
CAD: Canadian
CIHR: Canadian Institute for Health Research
COPD: Chronic obstructive pulmonary disease
COVID-19: Coronavirus-19
CRF: Case report form(s)
N: No
OCM: Outcome measure
PI: Primary investigator
PR: Pulmonary rehabilitation
RC: Research coordinator
RCT: Randomized controlled trial
REB: Research ethics board
Y: Yes
